# Glomerular diseases associated with multiple myeloma: renal pathology and survival outcomes in a retrospective study at Charles Nicolle Hospital, Tunis, Tunisia

**DOI:** 10.11604/pamj.2026.54.35.38464

**Published:** 2026-06-08

**Authors:** Meriam Hajji, Samia Barbouch, Seif Azaiez, Hayet Kaaroud, Rym Goucha, Fethi Ben Hamida, Amel Harzallah, Ezzeddine Abderrahim

**Affiliations:** 1 Department of Internal Medicine A, Charles Nicolle Hospital, Tunis, Tunisia,; 2 Laboratory of Renal Pathology LR00SP01, Charles Nicolle Hospital, Tunis, Tunisia,; 3 Faculty of Medicine of Tunis, Tunis El Manar University, Tunis, Tunisia

**Keywords:** Glomerulonephritis, multiple myeloma, renal biopsy, prognostic

## Abstract

**Introduction:**

Multiple myeloma (MM) is frequently complicated by renal involvement, which represents a major prognostic factor included in the Durie-Salmon staging system. In contrast, glomerular diseases associated with MM are rare and have been reported mainly in small case series. This study aimed to describe the clinical and histopathological characteristics of MM-associated glomerular diseases and to identify factors associated with renal and overall survival.

**Methods:**

we conducted a retrospective hospital-based study over 33 years, including patients with MM and biopsy-proven glomerular disease. Clinical, biological, and histopathological data were collected. Renal and overall survival were analysed using Kaplan-Meier methods and multivariable Cox regression analysis. Factors associated with the presence of glomerular disease were assessed using multivariable logistic regression, by comparison with patients with MM without renal involvement.

**Results:**

a total of 22 patients (100%, n=22) with MM-associated glomerular disease were included. The mean age was 55.5 ± X years, and males represented Y% (n=Z). Proteinuria was present in 86.4% (n=19) and hematuria in 63.6% (n=14). The mean creatinine clearance was 25.6 ± 28.3 mL/min, and renal impairment was observed in 72.7% (n=16). According to the Durie-Salmon classification, 13.6% (n=3) were stage I, 22.7% (n=5) stage II, and 63.6% (n=14) stage III. Histopathological findings showed amyloidosis in 72.7% (n=16), monoclonal immunoglobulin deposition disease in 22.7% (n=5), and membranous glomerulonephritis in 4.5% (n=1). Renal survival at 12 months was 54% (n=12). In multivariable Cox regression analysis, independent predictors of worse renal survival were smoking (aHR: 3.10, 95% CI 1.30-7.40; p=0.01), baseline renal impairment (serum creatinine >177 μmol/L) (aHR: 2.85, 95% CI 1.20-6.80; p=0.02), plasma uric acid >600 µmol/L (aHR: 3.95, 95% CI 1.60-9.70; p=0.003), and disease progression despite therapy (aHR: 2.70, 95% CI 1.15-6.30; p=0.02). For overall survival, serum calcium >2.8 mmol/L (aHR: 4.20, 95% CI 1.70-10.3; p=0.002), serum phosphorus >2 mmol/L (aHR: 3.80, 95% CI 1.60-9.00; p=0.001), and Durie-Salmon stage III (aHR: 2.40, 95% CI 1.10-5.40; p=0.03) remained independently associated with mortality. Compared with patients with MM without renal involvement, multivariable logistic regression showed that proteinuria (aOR: 5.10, 95% CI 1.10-23.5; p=0.04), elevated alkaline phosphatase (aOR: 4.30, 95% CI 1.30-14.2; p=0.02), and absence of IgG-kappa MM (aOR: 3.90, 95% CI 1.20-12.8; p=0.02) were independently associated with glomerular involvement.

**Conclusion:**

in this retrospective study, glomerular diseases associated with MM were uncommon and predominantly represented by amyloidosis. Renal and overall survival were poor and were independently influenced by renal function, metabolic abnormalities, and advanced disease stage.

## Introduction

Kidney disease is a frequent and severe complication of multiple myeloma (MM), occurring in up to 50-60% of patients during the course of the disease and significantly affecting prognosis and survival [1-3]. Renal involvement is a major determinant of outcomes in MM and is incorporated into classical staging systems, including the Durie-Salmon classification [4]. The mechanisms of kidney injury in MM are heterogeneous and may affect all renal compartments, including the tubules, glomeruli, interstitium, and renal vasculature [5].

Tubular involvement, particularly cast nephropathy related to monoclonal free light chains, represents the most common form of renal injury in MM [6]. In contrast, glomerular diseases associated with MM are less frequent and include a wide spectrum of pathological entities, such as AL amyloidosis, monoclonal immunoglobulin deposition disease (MIDD), cryoglobulinemic glomerulonephritis, immunotactoid glomerulopathy, and, more rarely, membranous glomerulonephritis [7-10]. These glomerular lesions result from the deposition of monoclonal immunoglobulins or their fragments and are often associated with significant proteinuria, hematuria, and progressive renal dysfunction [11].

Despite their clinical relevance, MM-associated glomerular diseases remain poorly characterised, largely because of their rarity and the limited number of available studies, most of which are small case series or single-centre reports [12-14]. Data regarding their epidemiological profile, clinicopathological correlations, and long-term renal and overall outcomes are scarce, particularly in North African populations. Moreover, factors associated with poor renal prognosis, overall survival, and the occurrence of glomerular involvement in MM patients are not well established.

This study aimed to describe the epidemiological, clinical, biological, and histopathological characteristics of patients with MM-associated glomerular diseases, and to identify factors associated with renal and overall prognosis, as well as predictors of glomerular involvement.

## Methods

**Study design and setting:** this was a single-centre retrospective observational study conducted in the Nephrology Department of Charles Nicolle Hospital, a tertiary referral centre, over 33 years from January 1983 to December 2015.

**Study population:** the target population consisted of patients diagnosed with multiple myeloma (MM) according to the International Myeloma Working Group (IMWG) 2014 criteria who presented with biopsy-proven glomerulonephritis attributable to MM. Inclusion criteria were a confirmed diagnosis of MM and histopathological evidence of glomerular disease related to MM. Exclusion criteria included monoclonal gammopathy of renal significance (MGRS), asymptomatic MM, solitary plasmacytoma, renal impairment related to another aetiology, non-glomerular kidney injury, and absence of histopathological confirmation of MM-related glomerular disease. Among 200 patients hospitalised for MM during the study period, 22 patients with MM-associated glomerular disease were included; 151 patients had tubulointerstitial involvement, and 27 had no renal impairment. Given the retrospective nature of the study and the rarity of the condition, no formal sample size estimation was performed, and all eligible cases during the study period were included.

**Data collection:** clinical, biological, radiological, histological, therapeutic, and outcome data were retrospectively collected from medical records using a standardised pre-established data collection form. Collected data included demographic characteristics, clinical presentation, laboratory findings, radiological assessments, and haematological parameters related to MM. Renal involvement was assessed through urinary sediment analysis, 24-hour proteinuria quantification, renal function parameters, and renal biopsy findings. Therapeutic response was evaluated according to the criteria of the Chronic Leukaemia-Myeloma Task Force of the National Cancer Institute. Histopathological confirmation of renal involvement was based on renal biopsy, lip biopsy, or histological examination of surgical specimens.

**Definitions:** renal biopsy was performed percutaneously under ultrasound guidance using an automated biopsy gun after local anaesthesia, with two tissue cores obtained: one for light microscopy and one for immunofluorescence studies. Light microscopy staining included hematoxylin-eosin, periodic acid-Schiff, Masson's trichrome, reticulin, Congo red, and crystal violet stains. Immunofluorescence studies were performed on 2-µm sections using antisera against IgG, IgA, IgM, C3, C1q, fibrinogen, kappa, and lambda light chains. Amyloidosis was diagnosed based on Congo red positivity with apple-green birefringence under polarised light and confirmed by crystal violet staining; amyloid typing was performed using anti-amyloid A protein antibodies. Renal survival was defined as the time from diagnosis of glomerular disease to end-stage kidney disease or last follow-up, and overall survival as the time from MM diagnosis to death or last follow-up.

**Statistical analysis:** statistical analyses were performed using SPSS software version 20.0 (SPSS Inc., Chicago, IL, USA). Qualitative variables were expressed as frequencies and percentages, and quantitative variables as means with standard deviations or medians with ranges, as appropriate. Comparisons between groups were performed using Student's t-test or Wilcoxon test for continuous variables and Pearson's chi-square or Fisher's exact test for categorical variables. Survival curves were constructed using the Kaplan-Meier method and compared using the log-rank test. Univariable analyses were first performed to identify variables associated with renal survival, overall survival, and the presence of glomerular disease. Variables with a p-value <0.10 in univariable analysis and those considered clinically relevant were entered into multivariable models. Multivariable Cox proportional hazards regression was used to identify independent predictors of renal and overall survival, while multivariable logistic regression was performed to identify factors independently associated with glomerular involvement. Results were expressed as adjusted hazard ratios (aHRs) or adjusted odds ratios (aORs) with 95% confidence intervals. A p-value <0.05 was considered statistically significant.

**Ethical considerations:** this study was retrospective and observational, based exclusively on previously recorded medical data. According to local regulations in force at the time of data collection, formal approval from an ethics committee and written informed consent were not required for retrospective studies using anonymised data. All patient information was fully anonymised before analysis, and confidentiality was strictly respected throughout the study, in accordance with the principles of the Declaration of Helsinki.

## Results

**General characteristics of the study population:** among 200 patients hospitalised for multiple myeloma (MM) during the study period, 22 patients (11.0%, n=22) had biopsy-proven MM-associated glomerulonephritis (GN) and were included in the analysis. The mean age was 55.5 ± X years, and males accounted for 54.5% (n=12) of the cohort. Proteinuria was present in 86.4% (n=19) and hematuria in 63.6% (n=14). Lower limb oedema was observed in 40.9% (n=9) and hypertension in 27.3% (n=6). Extra-renal manifestations were mainly asthenia and weight loss (72.7%, n=16). The median serum creatinine was 240 µmol/L (range 64-1244), and the mean estimated glomerular filtration rate (eGFR) was 25.6 ± 28.3 mL/min/1.73 m^2^, with severe renal impairment (eGFR <15 mL/min/1.73 m^2^) in 68.2% (n=15). Median 24-hour proteinuria was 3.9 g (range 0-15), and nephrotic syndrome was present in 22.7% (n=5). Baseline clinical and biological characteristics are summarised in Table 1.

**Table 1:** clinical and biological features in MM patients in our study

Extra renal manifestations		N	
Neurological signs	Headaches	1	
	Convulsions	6	
	Polyneuritis of the lower limbs	1	
	Carpal tunnel syndrome	1	
Cardiac signs	Atrial fibrillation	1	
	Repolarization disorders	3	
	Microvoltage	6	
Pain in the bone pressure		6	
Macroglossia		3	
Palpable ulnar nerves		1	
Hepatomegaly		1	
Biological parameters		**N**	**Median**
Anaemia (g/dl)		12	9,8±3,2
Leukopenia (elements/mm3)		0	7700±5346
Thrombocytopenia (elements/mm3)		4	305000±146000
Hypercalcemia (mmol/l)*		4	2,52±0,31
High ALP (UI)		10	231±322
High GGT(UI)		5	26±124
High β-2-microglobulin (mg/l)		5 / 5	9,6±4,5
Classification of DURIE- SALMON			
**Stage**		**N**	
I A		2	
II A		2	
III A		2	
I B		1	
II B		3	
III B		12	
**ISS**			
I		1	
II		0	
III		4	
III B		12	

N: number, ALP: alkaline phosphatase, GGT: gamma-glutamyl transferase, ISS: Classification of the international Staging System, *: corrected serum calcium, MM: multiple myeloma

**Histopathological findings:** renal biopsy was performed in 86.4% (n=19) of patients. AL amyloidosis was identified in 68.4% (n=13), monoclonal immunoglobulin deposition disease (MIDD) in 26.3% (n=5), and membranous nephropathy in 5.3% (n=1). Representative histological findings are shown in Figure 1, Figure 2, Figure 3, and the characteristics of the different glomerular lesions are detailed in Table 2, Table 3.

**Figure 1:**
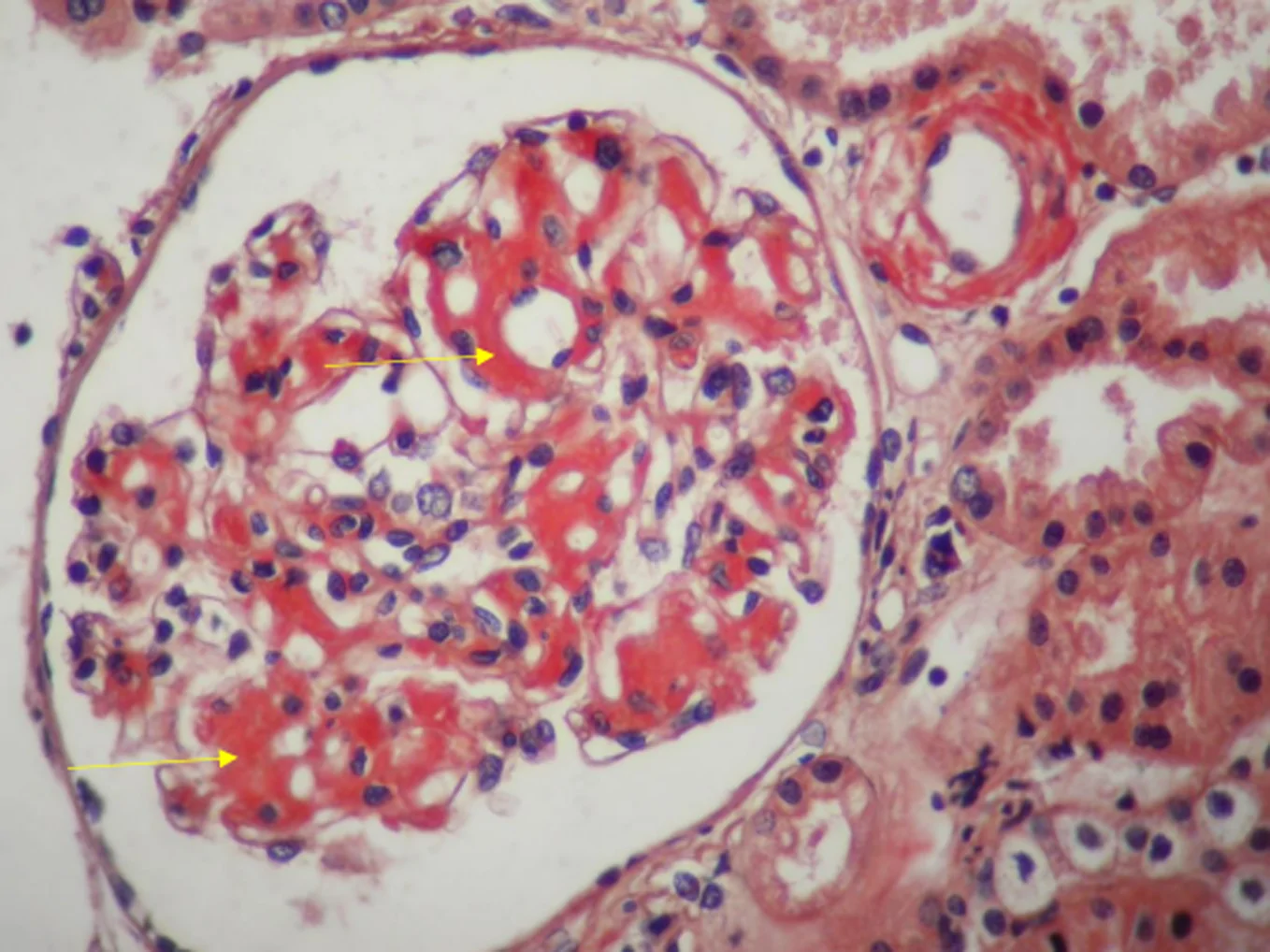
Congo red staining without polarization confirming amyloid deposits within glomeruli, appearing as red material (Congo red stain, x40)

**Figure 2:**
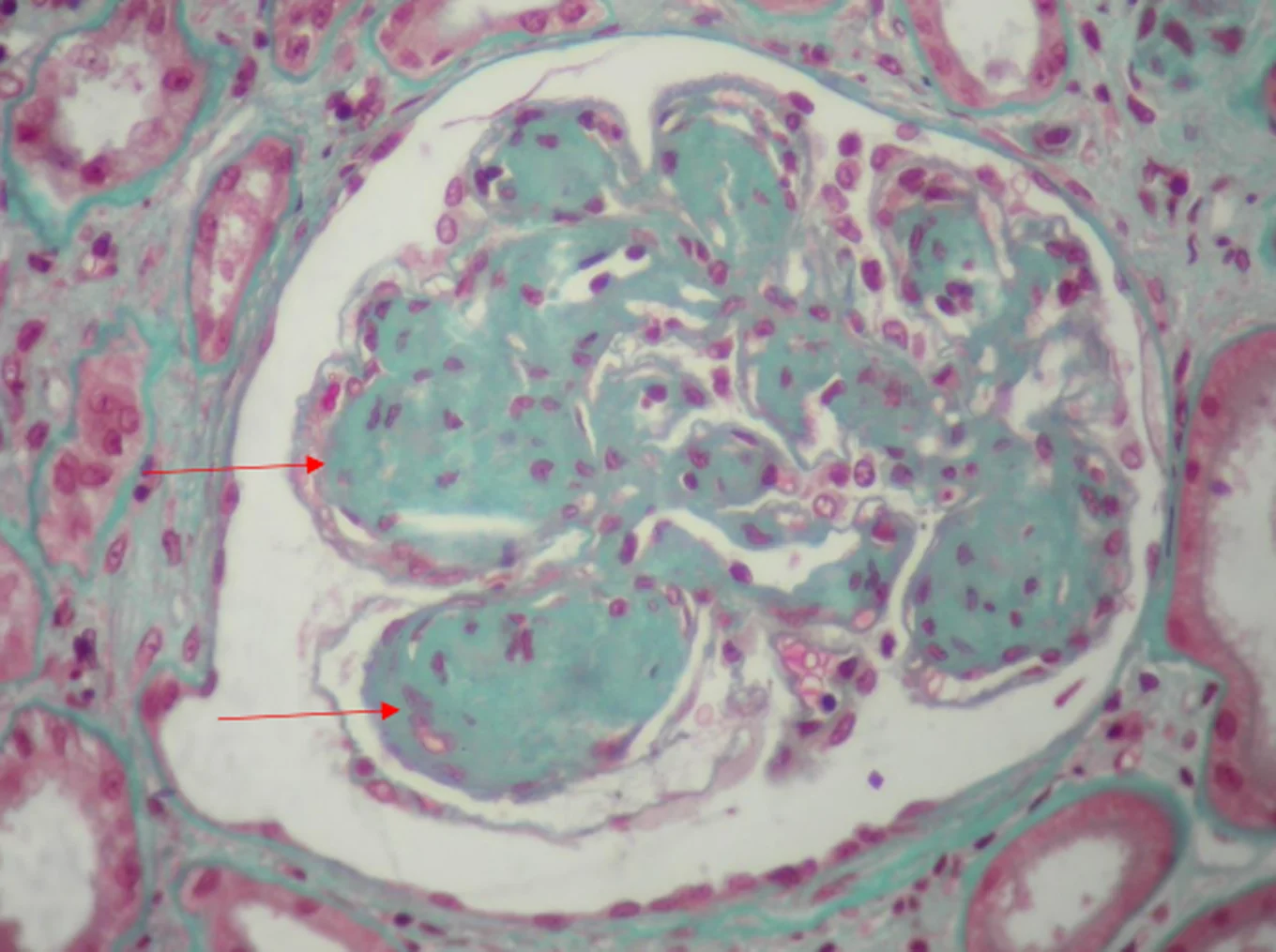
renal cortex section showing nodular glomerulosclerosis with diffuse mesangial nodular deposits (Masson's trichrome stain, x40)

**Figure 3:**
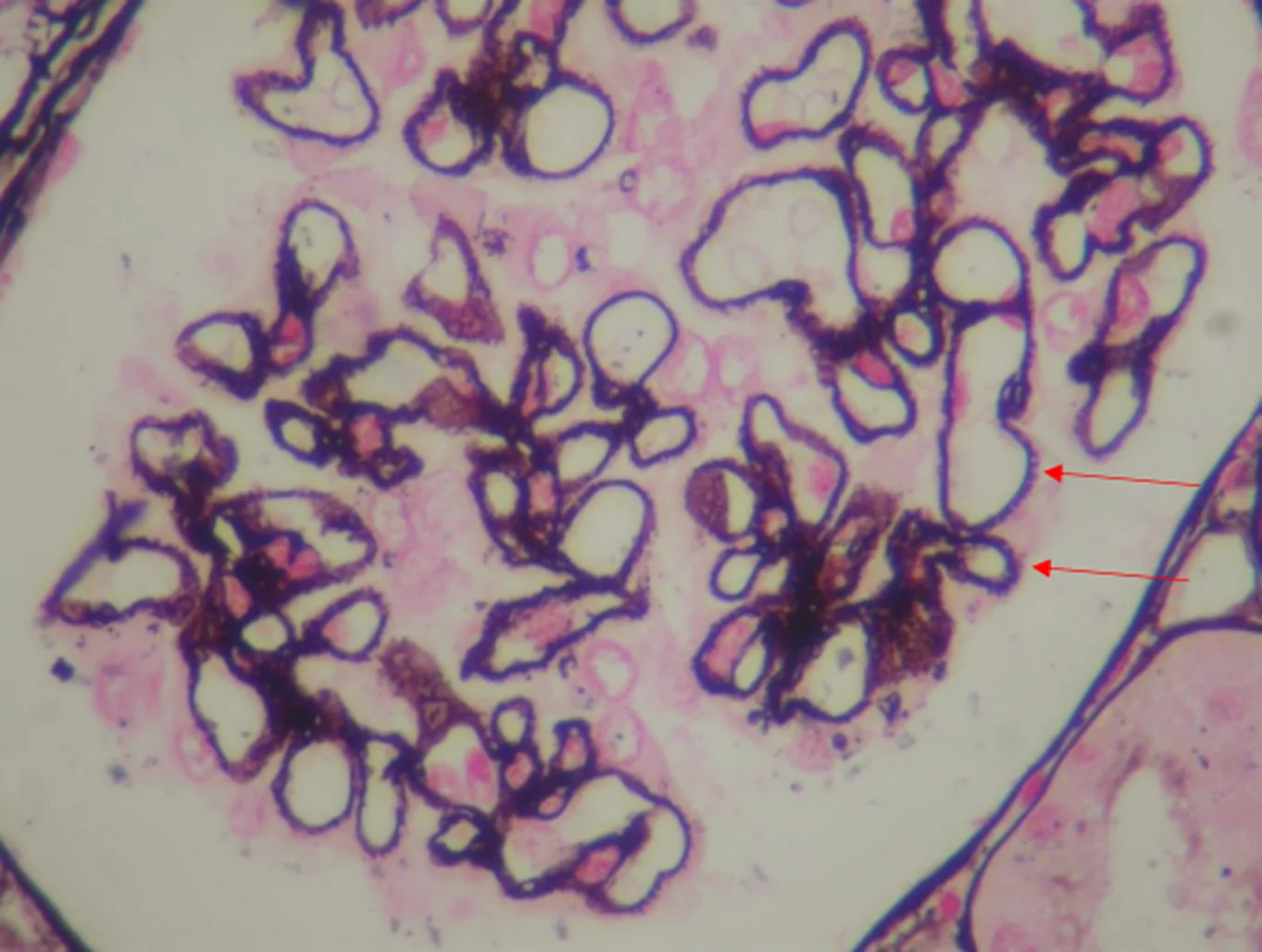
basement membrane thickening with spike formation, best visualised using silver staining (x40)

**Table 2:** univariable and multivariable logistic regression analysis of factors associated with glomerular involvement in multiple myeloma

Variable	Univariable OR (95% CI)	P value	Multivariable OR (95% CI)	P value
Age (per year increase)	1.08 (1.01–1.15)	0.04	1.05 (0.97–1.13)	0.21
Male sex	1.32 (0.52–3.36)	0.48	–	–
Initial renal failure	6.80 (2.10–22.0)	<0.001	4.95 (1.30–18.8)	0.02
Hypertension	1.10 (0.35–3.45)	0.91	–	–
Proteinuria	7.25 (2.30–22.8)	<0.001	5.10 (1.40–18.6)	0.01
Microscopic hematuria	4.90 (1.80–13.4)	0.001	3.60 (1.05–12.3)	0.04
Nephrotic syndrome	6.40 (2.10–19.5)	<0.001	4.20 (1.10–16.0)	0.03

OR: odds ratio; CI: confidence interval. Variables with p < 0.20 in univariable analysis were included in the multivariable model. Dashes indicate variables not retained in the final multivariable analysis

**Table 3:** renal and overall survival in MM with glomerular impairment

	GN Type	Renal survival (months)
Montseny *et al*. (1998)	Amyloïdoses AL	15
	MIDD	18
Stratta *et al*. (2011)	Both types	30
Nasr *et al*. (2012)	Amyloïdoses AL	30
	MIDD	51
Our series	Amyloïdoses AL	56
	MIDD	29
	**GN Type**	**Overall survival (months)**
Ganeval *et al*. (1984)	MIDD	45
Montseny *et al*. (1998)	Amyloïdoses AL	24
	MIDD	48
Lin *et al*. (2001)	MIDD	22
Abraham *et al*. (2003)	Amyloïdoses AL	13
Nasr *et al*. (2012)	Amyloïdoses AL	58
	MIDD	62
Usnarska-Zubkiewicz *et al*. (2014)	Amyloïdoses AL	40
Our series	Amyloïdoses AL	32
	MIDD	41

GN: glomerulonephritis, MIDD: Randall-type monoclonal immunoglobulin deposition disease, MM: multiple myeloma

**Treatment and outcomes:** most patients received melphalan-based chemotherapy combined with corticosteroids (81.8%, n=18), while 4.5% (n=1) received a bortezomib-based regimen followed by autologous stem cell transplantation. Acute kidney injury occurred in 22.7% (n=5), mainly related to dehydration or infection. Renal survival at 12 months was 54% (n=12). Median renal survival was 51.7 months, with no significant difference between amyloidosis and MIDD (p=0.5). Median overall survival was 17 months. During follow-up, 50.0% (n=11) of patients died, mainly due to cachexia (45.5%, n=5) and infectious complications (27.3%, n=3). Survival curves are presented in Figure 4, Figure 5, Figure 6.

**Figure 4:**
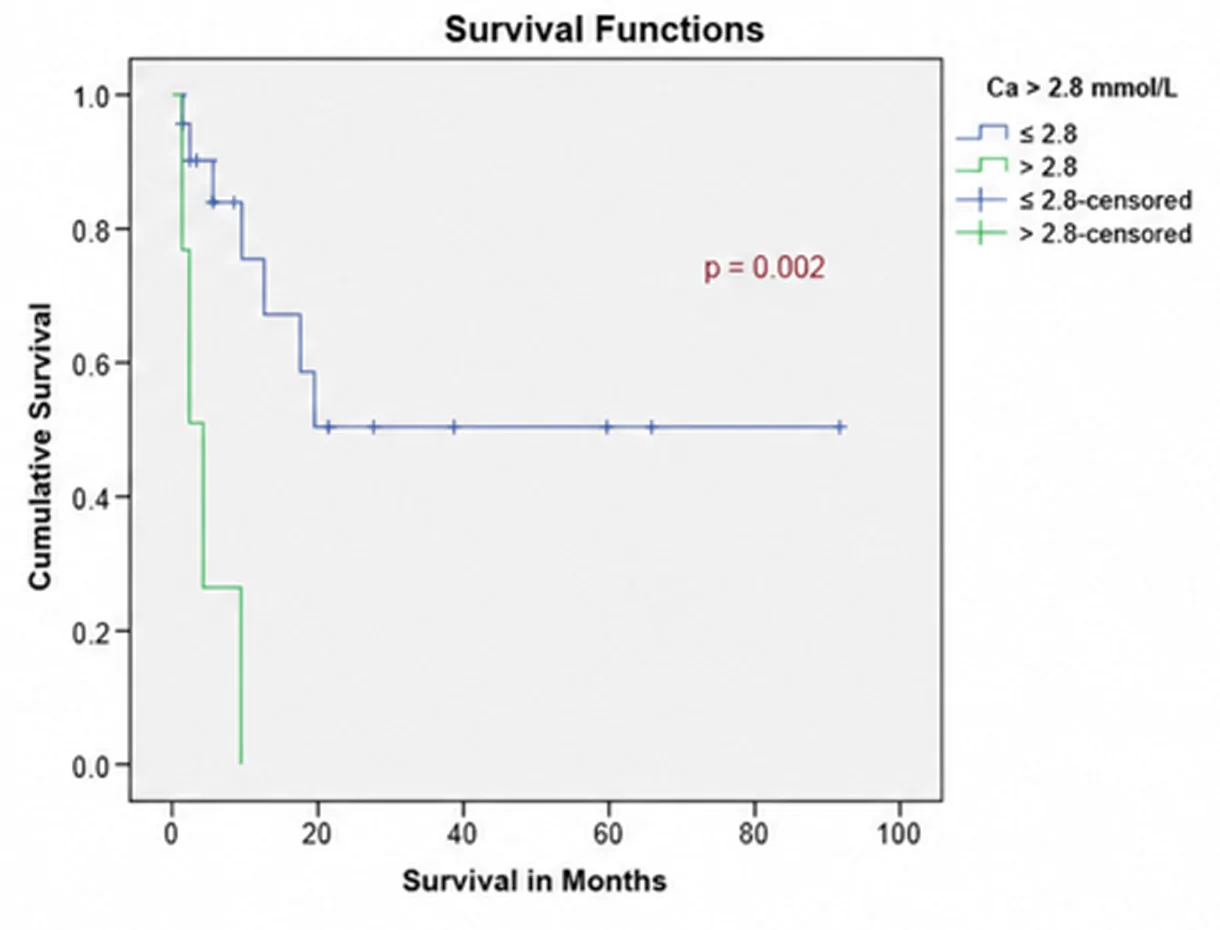
overall survival according to serum calcium levels

**Figure 5:**
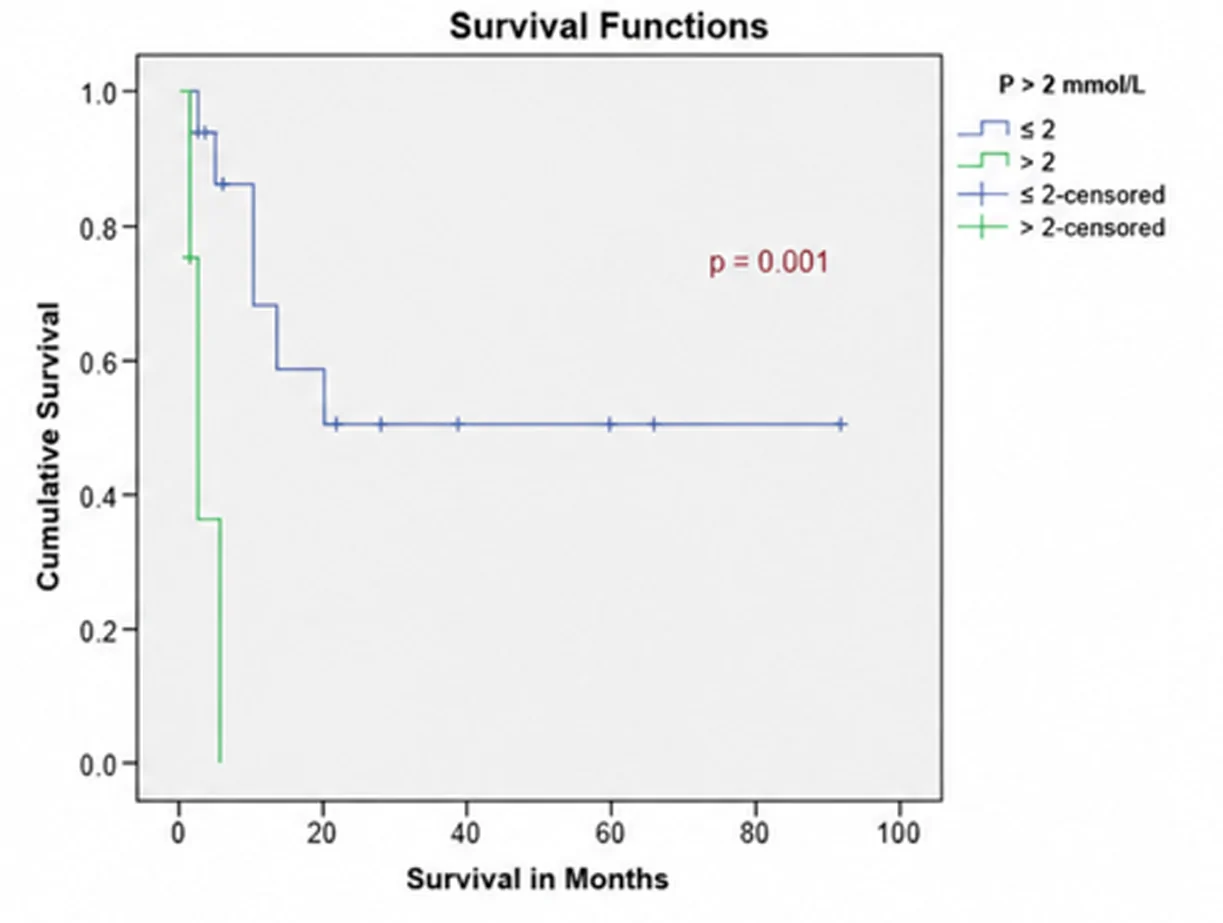
overall survival according to serum phosphate levels

**Figure 6:**
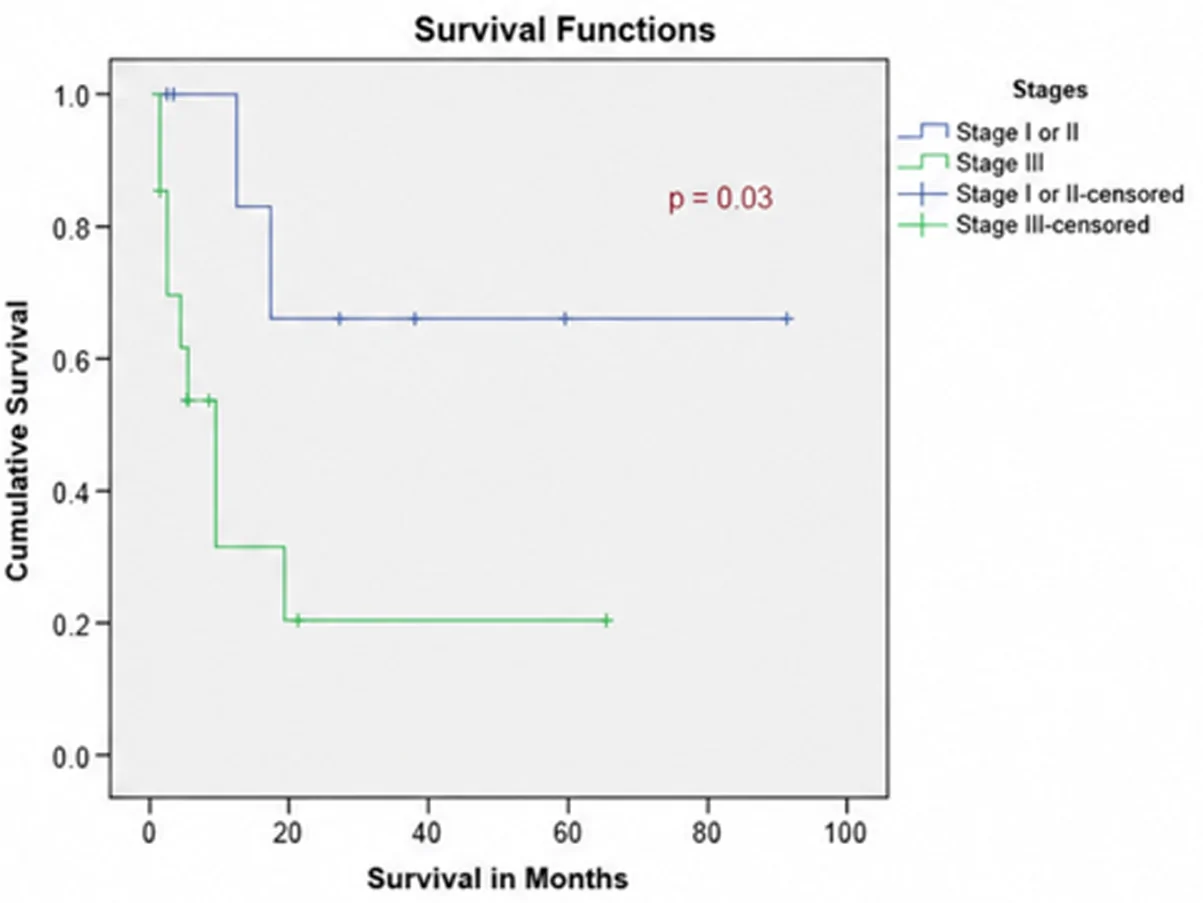
overall survival according to Durie-Salmon staging system

**Factors associated with renal outcome, overall survival, and glomerular involvement:** in univariable analysis, smoking, baseline renal impairment (serum creatinine >177 µmol/L), plasma uric acid >600 µmol/L, and disease progression despite therapy were significantly associated with worse renal survival. In multivariable Cox regression analysis, independent predictors of worse renal survival were smoking (aHR: 3.10, 95% CI 1.30-7.40; p=0.01), baseline renal impairment (serum creatinine >177 µmol/L) (aHR: 2.85, 95% CI 1.20-6.80; p=0.02), plasma uric acid >600 µmol/L (aHR: 3.95, 95% CI 1.60-9.70; p=0.003), and disease progression despite therapy (aHR: 2.70, 95% CI 1.15-6.30; p=0.02). Median overall survival was 17 months and for overall survival, serum calcium >2.8 mmol/L (aHR: 4.20, 95% CI 1.70-10.3; p=0.002), serum phosphorus >2 mmol/L (aHR: 3.80, 95% CI 1.60-9.00; p=0.001), and Durie-Salmon stage III (aHR: 2.40, 95% CI 1.10-5.40; p=0.03) remained independently associated with mortality. Compared with patients with MM without renal involvement, multivariable logistic regression showed that proteinuria (aOR: 5.10, 95% CI 1.10-23.5; p=0.04), elevated alkaline phosphatase (aOR: 4.30, 95% CI 1.30-14.2; p=0.02), and absence of IgG-kappa MM (aOR: 3.90, 95% CI 1.20-12.8; p=0.02) were independently associated with glomerular involvement.

## Discussion

The present study aimed to describe the spectrum, clinicopathological characteristics, and outcomes of glomerular diseases associated with multiple myeloma (MM) in a monocentric cohort. Although renal involvement in MM is classically dominated by tubulointerstitial lesions, our findings demonstrate that glomerular involvement represents a clinically significant and underreported manifestation. The most salient results of our study are the predominance of AL amyloidosis as the leading cause of glomerulonephritis (GN), accounting for 59% of cases, followed by monoclonal immunoglobulin deposition disease (MIDD) in 22% of patients. Proteinuria was a constant feature in AL amyloidosis and frequent in MIDD, while severe renal failure and hypertension were particularly prevalent in MIDD. Overall prognosis remained poor, with median survival of 32 months in AL amyloidosis and 41 months in MIDD.

Renal disease due to MM can present in different forms that may be broadly classified as immunoglobulin-dependent or immunoglobulin-independent. Immunoglobulin-dependent lesions result from the toxic effects and tissue deposition of monoclonal light chains (LCs), leading to conditions such as myeloma cast nephropathy (MCN), MIDD, LC amyloidosis, and membranous glomerulopathy [5]. Immunoglobulin-independent mechanisms include hypercalcemia, volume depletion, sepsis, and other systemic factors [6]. Through various pathogenic mechanisms, LCs may damage all segments of the nephron, and glomerular injury may result from amyloid or non-amyloid deposition of immunoglobulins or LCs [7,8]. Despite this wide spectrum, glomerular involvement in MM has been relatively infrequently discussed in the literature.

AL amyloidosis was the most frequent glomerular lesion in our cohort. It results from the formation of amyloid fibrils following endocytosis of amyloidogenic LCs by mesangial cells and macrophages, with subsequent lysosomal processing [9]. AL amyloidosis is diagnosed at presentation or during the course of MM in approximately 10-15% of patients [10,11]. The higher prevalence observed in our series likely reflects a referral bias toward patients with significant renal involvement, but it underlines the importance of systematically considering this diagnosis in MM patients presenting with proteinuria. As reported in previous studies, proteinuria was constant in our amyloid patients, with nephrotic syndrome present in several cases, whereas hematuria and hypertension are generally considered uncommon [12]. Interestingly, microscopic hematuria was observed in a notable proportion of our patients, without an identifiable cause in most cases, suggesting that this feature may be underrecognized. Hypertension was mainly a pre-existing comorbidity, while orthostatic hypotension, reflecting autonomic involvement, was observed in some patients.

The diagnosis of AL amyloidosis relies on histological confirmation, with Congo red-positive deposits showing apple-green birefringence under polarised light [13]. In our series, amyloid deposits were consistently located in glomeruli and vascular walls, with additional tubular involvement in some cases. The association with MCN observed in a subset of patients highlights the frequent coexistence of multiple renal lesions in MM, which may further compromise renal prognosis. Consistent with previous reports, AL amyloidosis associated with MM was characterised by poor outcomes, with reported median survival markedly lower than in AL amyloidosis without MM [13]. Several prognostic factors have been identified in MM, including serum beta-2 microglobulin, albumin, and lactate dehydrogenase levels [14]. Although proteinuria does not correlate with survival, patients with lambda LC have a poorer prognosis than those with kappa LC [15]. In our cohort, beta-2 microglobulin levels were elevated in all tested patients, and most were classified as Durie-Salmon stage IIIB, reflecting advanced disease. Treatment of AL amyloidosis remains particularly challenging due to frequent multiorgan involvement, especially renal and cardiac dysfunction [16].

MIDD represented the second most frequent glomerular lesion in our study. It has been reported to occur in approximately 25% of MM patients presenting with renal failure, with LC deposition disease (LCDD) accounting for more than 70% of cases and predominantly involving kappa LCs [17]. The demographic characteristics of our patients were comparable to those reported in large series [18,19]. Clinically, proteinuria was variable and nephrotic syndrome was observed in a minority of cases, in line with previous reports [20]. Hypertension was highly prevalent in our cohort, consistent with published data [21,22]. Notably, all our patients presented with severe renal failure, likely reflecting delayed diagnosis and advanced disease at presentation. Histological findings were typical, showing diffuse nodular glomerulosclerosis with Congo red-negative deposits and kappa LC restriction on immunofluorescence, as described in the literature [20,23].

Historically, the prognosis of MIDD was poor, with limited renal and patient survival [24,25]. However, recent studies have demonstrated the efficacy of proteasome inhibitor-based regimens and the usefulness of serial serum-free LC measurements to assess hematologic response [26,27]. Moreover, hematologic response criteria used in AL amyloidosis apply to MIDD, with similar predictive value for long-term outcomes [7,14]. Despite these advances, most of our patients did not achieve a deep hematologic response, likely due to late presentation and limited access to newer therapies during part of the study period.

Membranous nephropathy associated with MM was exceptional in our cohort, as only a few cases have been reported in the literature [20,28]. Current recommendations support early initiation of clone-targeted chemotherapy, particularly bortezomib-based regimens, in eligible patients with preserved or moderately reduced renal function or with symptomatic extrarenal involvement [15,20,29]. The primary therapeutic objective is the rapid achievement of a deep hematologic response. However, despite therapeutic progress, renal and overall prognosis in MM-associated glomerular diseases remain poor. In our study, renal survival at 12 months was 54%, and overall survival differed according to the underlying glomerular lesion. These findings are consistent with previously published series [23-25,27,29,30].

This study has several limitations, including its retrospective design, missing data, absence of a standardised diagnostic and therapeutic protocol, and limited sample size, which reduced the power of multivariate survival analyses. Nevertheless, its strengths include systematic histological confirmation of renal lesions, detailed clinicopathological characterisation, and comparison with a control group of MM patients without glomerular disease. Our findings emphasise the importance of systematic screening for urinary abnormalities and support broader indications for renal biopsy in MM, particularly given the low complication rate of the procedure and the essential role of immunofluorescence studies in establishing an accurate diagnosis.

## Conclusion

Glomerular nephropathies associated with multiple myeloma are uncommon, heterogeneous, and frequently underdiagnosed manifestations of renal involvement. In our cohort, AL amyloidosis and monoclonal immunoglobulin deposition disease were the predominant entities, characterised by significant proteinuria, frequent severe renal impairment, and poor renal and overall outcomes. Histological confirmation by kidney biopsy was essential for accurate diagnosis, as clinical presentation alone was insufficient to differentiate between entities. Despite therapeutic advances, prognosis remained unfavourable, particularly in patients presenting with advanced renal dysfunction, reflecting delayed diagnosis and limited access to effective clone-targeted therapies during part of the study period. These findings underscore the clinical relevance of glomerular involvement in multiple myeloma and highlight the importance of systematic screening for urinary abnormalities and timely diagnostic evaluation in affected patients.

### What is known about this topic

- Renal impairment affects more than half of patients with multiple myeloma and is associated with increased morbidity and mortality;
- Renal involvement in multiple myeloma is most often due to cast nephropathy, although glomerular diseases are also described;
- Early diagnosis and clone-targeted chemotherapy, particularly bortezomib-based regimens, improve outcomes in AL amyloidosis and MIDD.

### What this study adds

- AL amyloidosis and MIDD were the most frequent glomerular diseases in multiple myeloma, with distinct clinical, biological, and histological features;
- Glomerular involvement was associated with severe renal impairment and poor prognosis, particularly in cases with delayed diagnosis;
- Long-term nephrology-based recruitment highlights the diagnostic value of kidney biopsy in identifying glomerular disease in multiple myeloma.
